# A validation of the PAWPER XL-MAC tape for total body weight estimation in preschool children from low- and middle-income countries

**DOI:** 10.1371/journal.pone.0210332

**Published:** 2019-01-07

**Authors:** Mike Wells

**Affiliations:** Division of Emergency Medicine, Faculty of Health Sciences, University of the Witwatersrand, Johannesburg, South Africa; Kansas State University, UNITED STATES

## Abstract

**Importance:**

The PAWPER tape system is one of the three most accurate paediatric weight estimation systems in the world. The latest version of the tape, which does not rely on a subjective assessment of habitus, is the PAWPER XL-MAC method which uses length and mid-arm circumference (MAC) to estimate weight. It was derived and validated in a population in the USA and has not yet been fully validated in a population from a resource-limited setting.

**Objective:**

The objective of this study was to evaluate the performance of the PAWPER XL-MAC tape weight estimation system in a large dataset sample of children from resource-limited settings.

**Methods:**

This was a “virtual” study in which weight estimates were generated using the PAWPER XL-MAC tape and Broselow tape 2007B and 2011A editions in a very large open access dataset. The dataset contained anthropometric information of children aged 6 to 59 months from standardised nutritional surveys in 51 low- and middle-income countries. The performance of PAWPER XL-MAC method was compared with the Broselow tape and a new length- and habitus-based tape, the Ralston method.

**Main outcomes and measures:**

The bias of the weight estimation methods was assessed using the mean percentage error (MPE) and precision using the 95% limits of agreement (LOA) of the MPE. The overall accuracy was denoted by the percentage of weight estimates falling within 10% and 20% of actual weight (abbreviated as p10 and p20 respectively).

**Results:**

The MPE (LOA) for the PAWPER XL-MAC tape, the Broselow 2007B and 2011A and Ralston method were 1.9 (-15.3, 19.2), 5.4 (-15.9, 26.7), 7.7 (-13.3, 30.5) and -0.7 (-20.2, 19.3) respectively. The p10 and p20 for each method were 79.3% and 96.9% for the PAWPER XL-MAC tape, 64.3% and 91.0% for the Broselow tape 2007B, 55.5% and 85.9% for the Broselow tape 2011A and 67.4 and 94.0% for the Ralston method respectively. The PAWPER XL-MAC system was statistically significantly more accurate than the Broselow tape 2011A, the Broselow tape 2007B and the Ralston method. The relative difference in accuracy (p10) was 43% (odds ratio 4.4 (4.4, 4.5), p<0.001), 23% (odds ratio 2.9 (2.8, 2.9), p<0.001) and 18% (odds ratio 1.8 (1.8, 1.8), p<0.001) compared to each method, respectively.

**Conclusions and relevance:**

The PAWPER XL-MAC tape performed well in this study and was statistically significantly more accurate than both the Broselow tape editions and the Ralston method. This difference was substantial and clinically important. The tape did not perform as well at extremes of habitus-type, however, and might benefit from recalibration.

## Introduction

### The importance of weight estimation

There are two sets of circumstances under which a child’s bodyweight must be estimated: during emergency care when a child cannot be weighed even if a scale is available and in resource-limited settings where a scale might not be available at all [1, 2]. In both these examples, a weight estimation system that could estimate weight accurately would be ideal to allow for the correct calculation of drug doses [3].

The PAWPER tape is one of the three most accurate weight estimation systems in the world today, along with the Mercy method and parental estimates of weight [4, 5]. The PAWPER tape system and the Mercy method are both dual length- and habitus-based methods which have consistently been shown to be more accurate than one-dimensional systems. The PAWPER tape has also proven to be accurate both in high-income countries as well as low- and middle-income countries [5, 6].

### The PAWPER tape systems

**Fig 1:**
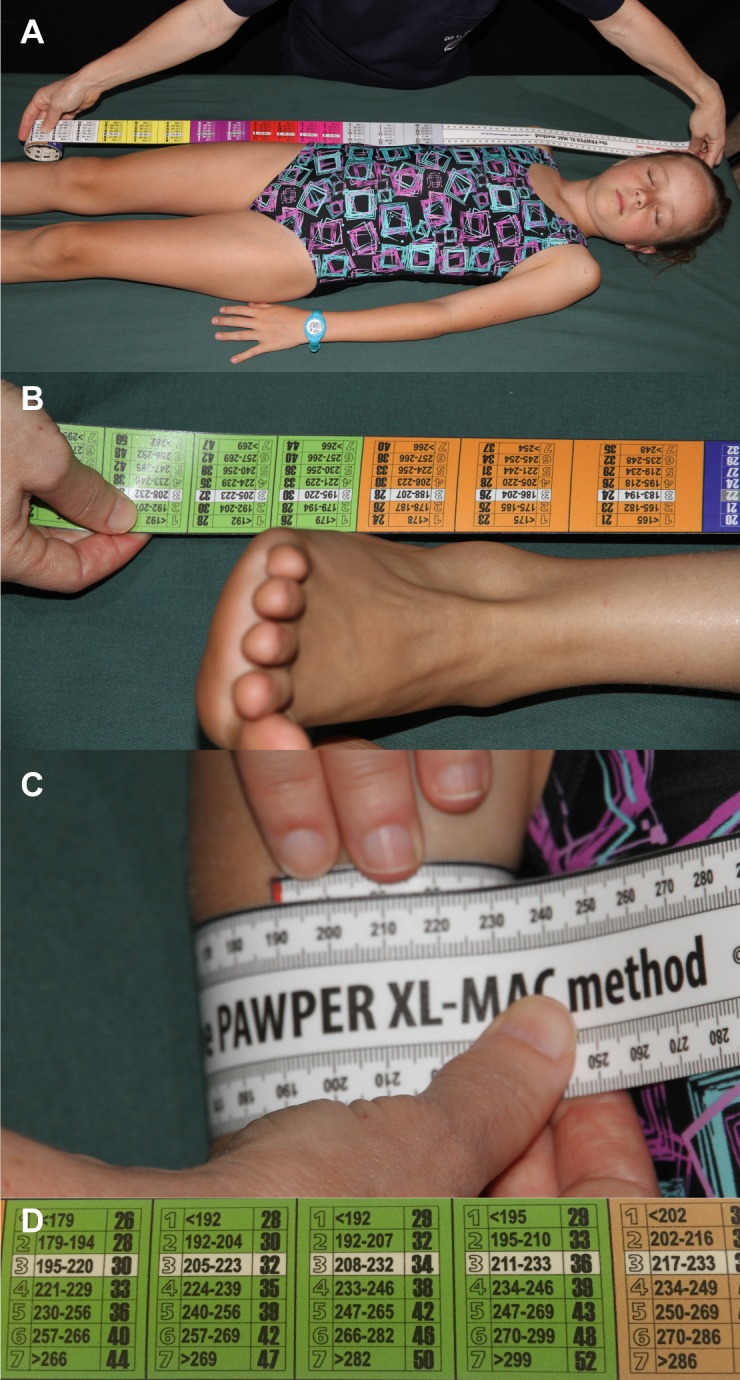
The PAWPER XL-MAC tape system. There are three steps to the use of the PAWPER XL-MAC tape. The first step is to measure the length of the child from his/her head to his/her heel (Panels A and B). The user thus identifies into which length segment the child falls. The second step is to measure the child’s mid-arm circumference using the tape (Panel C). The final step is to read off the estimated weight in the appropriate length segment based on the measured mid-arm circumference (Panel D). The user should be familiar and well-practised with the tape before using it in a resuscitation situation.

There are three versions of the PAWPER tape system: the original PAWPER tape (developed in 2009), the PAWPER XL tape (developed in 2014) and the PAWPER XL-MAC tape (developed in 2016)—see Fig 1 for a description of the PAWPER XL-MAC tape system [3, 7, 8]. The original PAWPER tape and the PAWPER XL tape both make use of body length and a visual assessment of habitus to allow a weight estimate to be read directly off the tape [7]. The PAWPER XL tape is longer than the original tape (180cm vs 145cm) and has additional capabilities to produce weight estimates in obese children (seven habitus categories vs five) [3]. The PAWPER XL-MAC tape is a completely objective system which makes use of mid-arm circumference (MAC) to define habitus instead of relying on a visual assessment of habitus [8]. Each length-segment of the tape contains MAC cut-off values which define the habitus category and allow the weight to be read directly off the tape.

The PAWPER XL-MAC tape was developed and validated using a combined National Health and Nutrition Examination Survey (NHANES) survey dataset from the USA [8]. Although the tape performed very well in children of all weight categories, it would be of great value to further evaluate the system in a population of much younger children from low- and middle-income countries.

The Ralston tape was designed following a study using this dataset [9]. It is essentially a (still theoretical) device with three side-to-side tapes: one to estimate the weight of “normal” children (MAC>125mm), one for “moderately wasted” children (MAC from 115 to 125mm) and one for “severely wasted” children (MAC<115mm). It was shown to be more accurate than the Broselow tape but has not been compared to other contemporary dual length- and habitus-based systems.

### Objectives

The primary objective of this study was to evaluate the accuracy of the PAWPER XL-MAC system in this large dataset of children from low- and middle-income countries. The secondary objective was to compare the performance of the PAWPER XL-MAC system with that of the Broselow tape (the existing “gold standard”) and the tape proposed by Ralston *et al* [9].

## Methods

### Study design

This was a retrospective, observational, “virtual” weight estimation study (a virtual study is one in which weight estimations are calculated from a database of anthropometric measurements, rather than a study in which a weight estimation system is directly applied to an individual child). The performance of the PAWPER XL-MAC system was evaluated using data from a very large, recently-published, open-access dataset [9]. This dataset is comprised of anthropometric data for children aged 6 to 59 months derived from growth studies conducted in multiple low- and middle-income countries.

### Dataset

The dataset is available at https://doi.org/10.6084/m9.figshare.6026504.v1. A full description of the dataset can be found in the article by Ralston *et al* from which it was obtained [9]. The dataset contains data from 2,434 nutritional anthropometric surveys from 51 low- and middle-income countries. The data was collected over a period of 25 years, from August 1992 to May 2017 and contains information on 1,717,172 children aged from 6 to 59 months. The number of data points differed slightly from the study by Ralston *et al* as some of the shared data required permissions from the United Nations High Commissioner for Refugees and was not included in this study (83,150 children). The data included the country of origin, year of collection, sex, measured weight, length (or height), mid-arm circumference, weight-for-age Z-score, height-for-age Z-score and weight-for-age Z-score. The data collection methodology was consistent across the surveys. Weight, height, and MAC measurements were standardized and performed by teams of trained researchers.

### Ethics

Ethical approval and informed consent was obtained for each individual surveys as described by Ralston *et al* [9]. The source data were only collected after ethical approval was obtained from locally responsible ethics committees. When no such ethics committees were functioning, ethical approval was acquired from the institutional review bodies of the non-governmental organization which collected the data. In addition, permissions were obtained from the local ministries of health and, where appropriate, other governmental organizations.

Participation in the surveys was strictly voluntary. In all surveys, the consent procedure was approved by institutional review boards and informed consent was obtained from the primary caregiver of the child. Identifying data were removed before the dataset was made available online.

The image of the child model in Fig 1 was included with written informed consent (as outlined in PLOS consent form) to publish this illustration.

### Data generation

Although the weight-for-age, height-for-age and weight-for-height z-scores were available in the dataset, the BMI and BMI-for-age Z-scores were not. These were calculated for each child using a custom-designed spreadsheet formula based on the WHO BMI-for-age growth chart data.

PAWPER XL-MAC weight estimations were generated from the dataset using the available measurements of length (or height) and mid-arm circumference for each child. The cut-off values of mid-arm circumference for each length-division of the PAWPER XL-MAC tape have been published previously [8]. These values were incorporated into an excel formula which then automatically calculated the estimated weights. The excel formula can be found in the Supplementary material (S1 file). In order to provide a standard against which to compare the PAWPER XL-MAC system, a similar process was followed to obtain weight estimations using two versions of the Broselow tape (the 2007B and 2011A editions), using the length data only. The length-segment data for the Broselow tape was obtained from a recent systematic review on the tape [10]. The data on the performance of the Ralston method was obtained from the paper in which it was described [9].

### Data analysis

The data analysis followed the recommended methods for weight estimation studies which focus on evaluating the bias, precision and overall accuracy of the weight estimation systems [5]. Bias was determined using the mean percentage error (MPE), shown in Formula 1 below.

$$Percentage\;error=100\times \frac{Estimated\;weight-Actual\;weight}{Actual\;weight}$$ (1)

A negative value would thus be indicative of an underestimation of weight. The precision was determined using the Bland & Altman 95% limits of agreement of the MPE as well as the root mean square percentage error (RMSPE), shown in Formulae 2 and 3 below.

Limitsofagreement=MPE±1.96×standarddeviation (2)

where MPE represents mean percentage error.

$$Root\;mean\;square\;percentage\;error=\frac{\Sigma \sqrt{{PE}^{2}}}{n}$$ (3)

where PE represents percentage error and n the sample size.

Percentage error calculations were preferred over absolute values (in kg). The true, measured weights ranged from 3.3kg to 25kg in the dataset, almost a 10-fold difference in weight. The implication of an error of 1kg is thus significantly different for a 3kg infant compared to a 25kg 5-year-old child. Therefore, the mean bias and limits of agreement (in kg) of untransformed or unscaled data is not statistically or clinically useful. Either logarithmically transformed data or percentage error data is more useful but percentage error data is more intuitive and easier to interpret. The overall accuracy of each weight estimation system was determined by calculating the proportion of weight estimations falling within 10% and 20% of actual, measured weight (p10 and p20 respectively).

### Outcome measures

The overall accuracy (represented by the p10 and p20 data) was used as the primary outcome measure as it best reflects the global performance of the weight estimation systems. It also most closely reflects the implications of the weight estimation on drug dosing accuracy. An acceptable outcome, based on previous studies, was a p10 of 70% and a p20 of 95% [5]. While the measures of bias and precision were evaluated, they are more statistically useful for refining and calibrating a weight estimation system and offer less intuitive information than overall accuracy.

### Subgroup analyses

The data was analysed according to subgroups of age, weight and BMI-for-age z-score. The age subgroups used were 6 to 12 months, 13 to 24 months, 25 to 36 months, 37 to 48 months and 49 to 59 months. The weight subgroups were ≤10kg, 10.1 to 15kg and >15kg. The BMI-for-age subgroups used were Z≤-2.0 (underweight), -2.0<Z≤-1.4 (thin), -1.4<Z<1.4 (“normal” weight), 1.4≤Z<2.0 (overweight) and Z≥2.0 (obese). Since BMI-for-age subgroup data was not available for the Ralston method, subgroup analyses and comparisons were performed using their published weight-for-height data. The consistency of the outcomes was also evaluated across the regions represented in the dataset.

### Comparisons between systems

In order to facilitate comparisons of accuracy between the PAWPER and Ralston methods, especially in subgroups, it was necessary to impute p10 and p20 data for the Ralston method. This was done by calculating these data using Formula 4 below. This formula is accurate in normally distributed data, as was true in this case.

$$p10=100\times \int_{x-10}^{x+10}f(x,\mu ,\sigma )=\frac{1}{\sqrt{2\pi \sigma }}\times e^{-(\frac{{\left(x-\mu \right)}^{2}}{{2\sigma }^{2}})}$$ (4)

where p10 represents the proportion of weight estimates falling within 10% of actual weight, *x* is the mean actual weight of the sample, μ is the mean estimate error and σ is the standard deviation of the mean estimate error.

Where statistical comparisons between the weight estimation systems were considered necessary, the paired t-test was used for comparisons of MPE and RMSPE and the McNemar test was used for paired comparisons of p10 and p20. A difference of more than 10% between any parameter was considered to be clinically or operationally important. Given the very large size of the dataset, and the use of multiple statistical tests to compare weight estimation methods, a 0.1% significance level (p<0.001) was used throughout to denote statistical significance and reduce the likelihood of type I error. The effect sizes were quantified using odds ratios with 95% confidence intervals.

### Software

Microsoft excel (Microsoft Excel for Mac version 16.14.1) and Graphpad Prism (GraphPad Prism version 8.00 for Mac, GraphPad Software, La Jolla California USA, www.graphpad.com) were used for all data management and statistical analysis.

## Results

A total of 1,717,172 children in the dataset had data available to produce a weight estimation by the PAWPER XL-MAC system as well as two Broselow tape editions and were included in the study.

### Demographic data

A description of the demographic data of the sample population from the dataset can be found in Table 1.

**Table 1:** Basic demographic information of the children in the dataset.

	N (%)	
**Children total**	1,717,172 (100%)	
**Boys (%)**	868,500 (50.6%)	
**Girls (%)**	848,672 (49.4%)	
	**Mean (SD)**	**Median (IQR)**
**Age (months)**	31.3 (15.2)	30 (18, 44)
**Weight (kg)**	11.2 (2.8)	11.1 (9.0, 13.2)
**Height (cm)**	85.4 (11.6)	85.1 (76.1, 94.3)
**MAC (cm)**	14.2 (1.3)	14.2 (13.3, 15.1)
**BMI (kgm**^**-2**^**)**	15.2 (1.6)	15.1 (14.2, 16.2)
**BMI-for-age (Z-score)**	-1.0 (1.5)	-0.9 (-1.9, 0.0)
**Height-for-age (Z-score)**	-1.5 (1.5)	-1.5 (-2.5, -0.6)
**Weight-for-age (Z-score)**	-1.3 (1.2)	-1.3 (-2.0, -0.6)
**Weight-for-height (Z-score)**	-0.6 (1.2)	-0.6 (-1.4, 0.1)

The mean and standard deviation (SD) as well as the median and interquartile range (IQR) are shown.

The distribution of children according to WHO weight-for-height, height-for-age and weight-for-age Z-score classification is shown in Table 2. The majority of children were classified as “normal” (i.e. without severe malnutrition, major wasting or stunting), but there were sufficient numbers of children in each category to test the weight estimation systems. According to the BMI-for-age classification, 23.1% of the children were underweight (Z≤-2.0), 13.2% were “thin” (-2.0<Z≤-1.4), 59.7% were of normal weight (-1.4<Z<1.4), 2.5% were overweight (1.4≤Z<2.0) and 1.5% were obese (Z≥2.0).

**Table 2:** Children classified according to normal and abnormal growth or body weight.

	Z-score categories		
	Z≥-2.0 N (%)	-2.0<Z≤-3.0 N (%)	Z<-3.0 N (%)
**Weight-for-height**	1,514,325 (88.2%)	157,807 (9.2%)	45,040 (2.6%)
**Height-for-age**	1,092,965 (63.6%)	362,368 (21.1%)	261,839 (15.2)
**Weight-for-age**	1,275,437 (74.3%)	312,733 (18.2%)	129,002 (7.5%)

Children with a weight-for-height Z-score of ≥-2.0 are classified as having “no wasting”, those with a Z-score -2<Z≤-3 as having “moderate wasting” and those with a Z-score of <-3.0 are classified as having “severe wasting”. Similarly, children with a weight-for-age Z-score of ≥-2.0 are classified as having “normal weight”, those with a Z-score -2<Z≤-3 as being “moderately underweight” and those with a Z-score of <-3.0 are classified as being “severely underweight”. Children with a height-for-age Z-score of ≥-2.0 are classified as having “no stunting”, those with a Z-score -2<Z≤-3 as having “moderate stunting” and those with a Z-score of <-3.0 are classified as having “severe stunting”.

**Fig 2:**
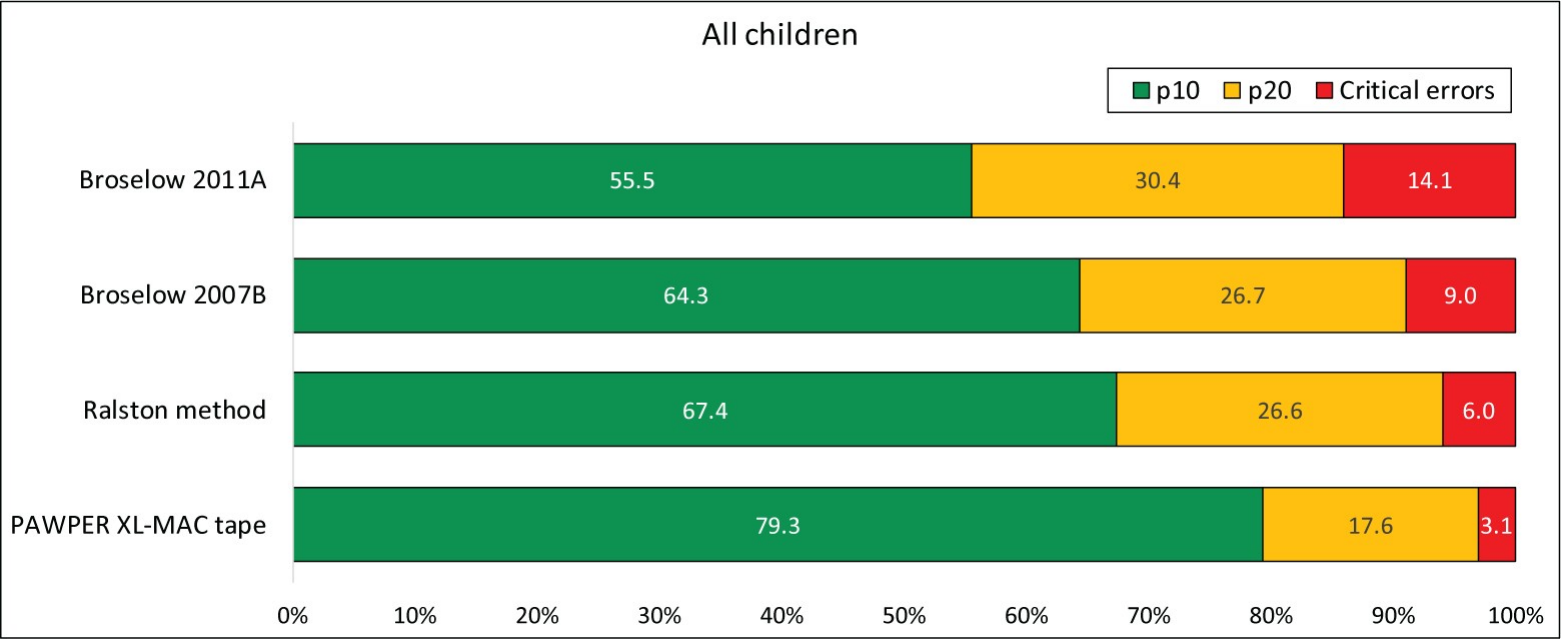
Analyses of the overall accuracy of the weight estimation systems. The chart shows the proportion of weight estimations falling within 10% and 20% of actual weight (p10 and p20 respectively) as well as the proportion of critical weight estimation errors (>20% error). The data for the Ralston method was obtained from the published study [9]. The McNemar test was significant at the p<0.001 level for every comparison of p10 and p20. The PAWPER XL-MAC method’s p10 beached the 10% improvement criterion when compared with the other methods. The p10 of the Broselow 2011A was clinically inferior to all other methods.

**Fig 3:**
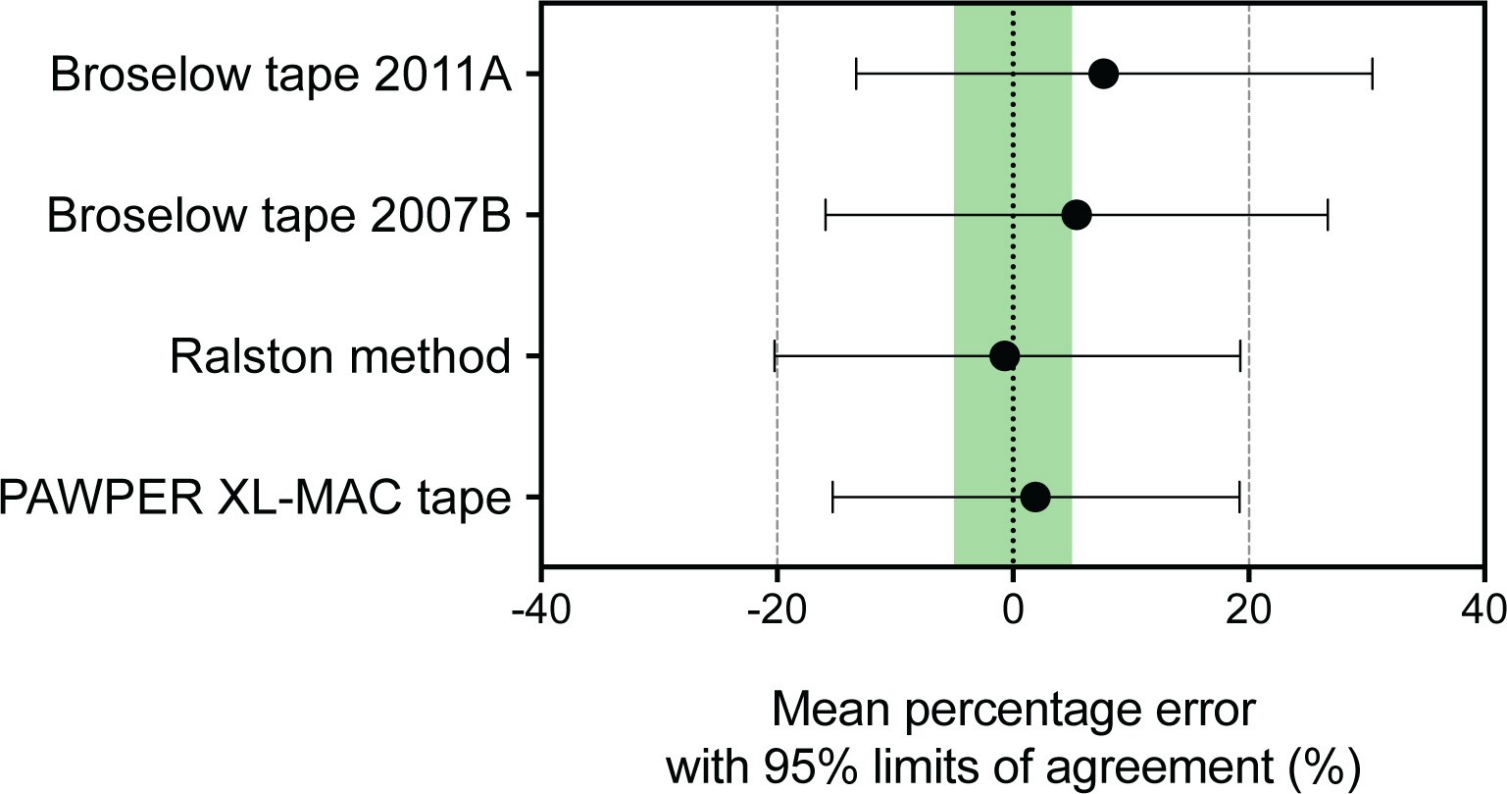
Analyses of the bias and precision of the weight estimation systems. The forest plot shows the overall bias (the black circles indicate the mean percentage error), as well as the precision (the whiskers indicate the Bland & Altman 95% limits of agreement) for each system. The green shaded area denotes an acceptable MPE (within ±5%), while the dashed lines indicate an acceptable range for the 95% LOA (within ±20%). The paired t-test was significant at the p<0.001 level for every comparison of MPE and RMSPE. The MPE of the PAWPER XL-MAC and Ralston methods were clinically superior to the Broselow tape methods. The precision (quantified using the RMSPE) of the PAWPER XL-MAC method was clinically superior to all other methods.

The overall results of the assessment of the performance of the three weight estimation systems are shown in Figs 2 and 3. The PAWPER XL-MAC system was statistically significantly more accurate than the Broselow tape 2011A, the Broselow tape 2007B and the Ralston method. The absolute and relative differences in accuracy (p10) were 24% and 43% (odds ratio 4.4 (4.4, 4.5), p<0.001) compared to the Broselow tape 2011A, 15% and 23% (odds ratio 2.9 (2.8, 2.9), p<0.001) compared to the Broselow tape 2007B and 12% and 18% (odds ratio 1.8 (1.8, 1.8), p<0.001) compared to the Ralston method. These differences were all clinically relevant. The details of the statistical analyses for the subgroup comparisons can be found in the Supplementary material (S1 Table).

**Fig 4:**
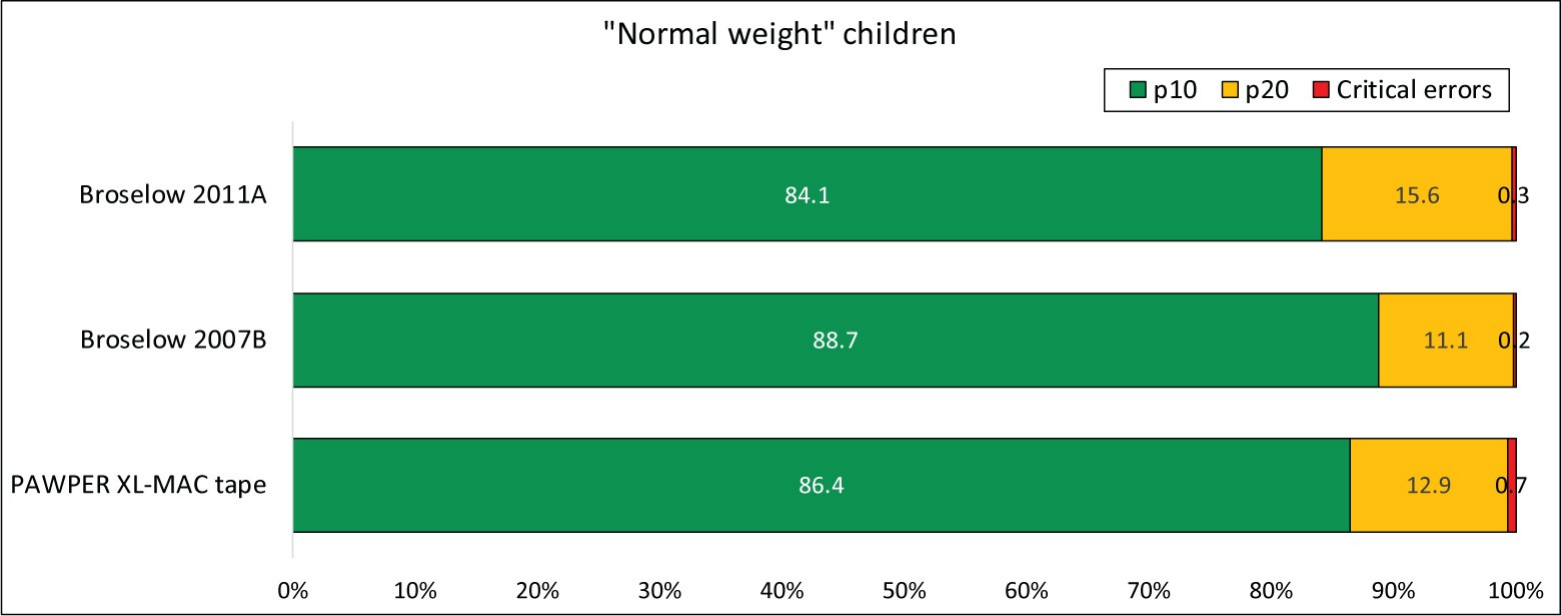
Accuracy of the three systems in “normal weight” children. The chart shows the proportion of weight estimations falling within 10% and 20% of actual weight (p10 and p20 respectively) as well as the proportion of critical weight estimation errors (>20% error) for children with a BMI-for-age Z-score between -1.4 and 1.4. This subgroup data was not available for the Ralston method as it information was not presented in the original publication. The McNemar test was significant at the p<0.001 level for every comparison of p10 and p20. However, the differences were not clinically important.

**Fig 5:**
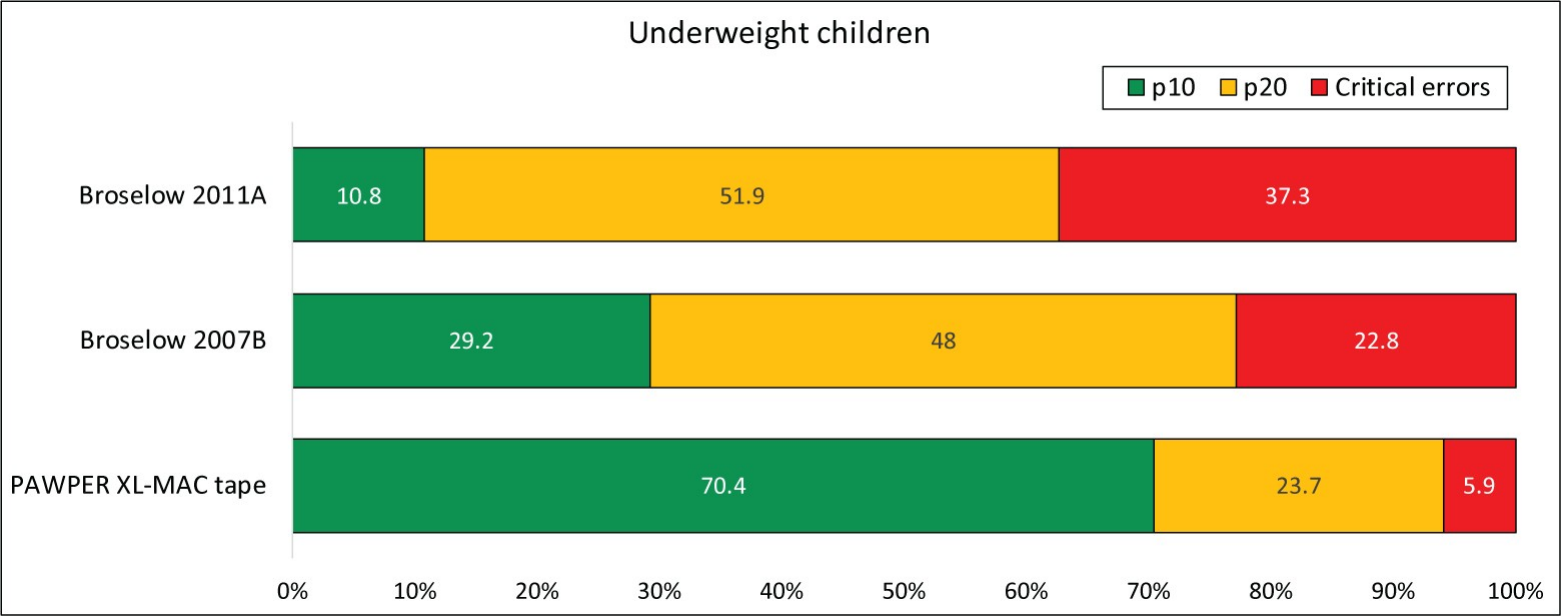
Accuracy of the three systems in underweight children. The chart shows the proportion of weight estimations falling within 10% and 20% of actual weight (p10 and p20 respectively) as well as the proportion of critical weight estimation errors (>20% error) for children with a BMI-for-age Z-score less than -1.4. This subgroup data was not available for the Ralston method as it information was not presented in the original publication. The McNemar test was significant at the p<0.001 level for every comparison of p10 and p20. The PAWPER XL-MAC method p10 and p20 were clinically superior to both versions of the Broselow tape and the Broselow 2007B was clinically superior to the Broselow 2011A.

**Fig 6:**
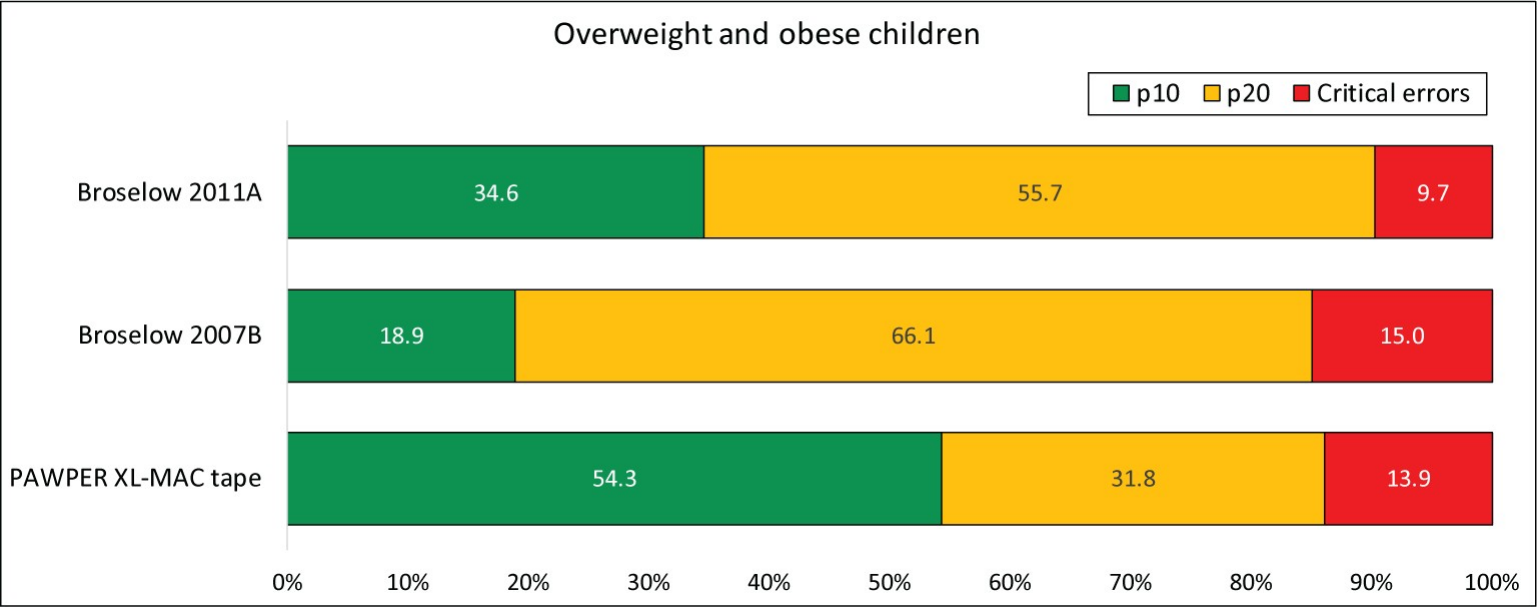
Accuracy of the three systems in overweight and obese children. The chart shows the proportion of weight estimations falling within 10% and 20% of actual weight (p10 and p20 respectively) as well as the proportion of critical weight estimation errors (>20% error) for children with a BMI-for-age Z-score greater than 1.4. This subgroup data was not available for the Ralston method as it information was not presented in the original publication. The McNemar test was significant at the p<0.001 level for every comparison of p10 and p20. The PAWPER XL-MAC method p10 and p20 were clinically superior to both versions of the Broselow tape. The p10 of the Broselow 2011A was clinically superior to the Broselow 2007B, but the p20 of the Broselow 2007B was clinically superior to the Broselow 2011A.

Figs 4, 5 and 6 show the accuracy outcome data for each system according to BMI-for-age weight status (normal weight, underweight and overweight/obese).

Tables 3–6 show the results of the subgroup analyses of the performance of the weight estimation systems according to sex, age, weight and weight status.

**Table 3:** Weight estimation performance by subgroups of sex.

			MPE (LOA)	RMSPE	p10	p20
**PAWPER XL-MAC**	**All**	1,717,172	1.9 (-15.3, 19.2)	6.9 (5.9)	79.3	96.9
	**Boys**	868,500 (50.6%)	1.2 (-16.0, 18.3)	6.7 (5.6)	80.4	97.3
	**Girls**	848,672 (49.4%)	2.7 (-14.7, 20.2)	7.1 (5.9)	78.1	96.5
**Broselow 2007B**	**All**	1,717,172	5.4 (-15.9, 26.7)	9.4 (7.7)	64.3	91.0
	**Boys**	868,500 (50.6%)	3.6 (-16.5, 25.5)	8.9 (7.3)	67.0	92.5
	**Girls**	848,672 (49.4%)	5.6 (-15.3, 28.1)	10.0 (7.7)	61.6	89.5
**Broselow 2011A**	**All**	1,717,172	7.7 (-13.3, 30.5)	11.2 (8.6)	55.5	85.9
	**Boys**	868,500 (50.6%)	6.7 (-13.9, 29.3)	10.5 (8.2)	58.7	88.0
	**Girls**	848,672 (49.4%)	8.8 (-12.7, 31.9)	11.9 (8.6)	52.1	83.7
**Ralston method**	**All**	1,800,322	-0.7 (-20.2, 19.3)	Not done	67.4	94.0
	**Boys**	No data				
	**Girls**	No data				

No subgroup data was available for the Ralston method. The subgroup analyses by sex showed the same statistical significance outcomes as for the whole population.

**Table 4:** Weight estimation performance by subgroups of age.

			MPE (LOA)	RMSPE	p10	p20
**PAWPER XL-MAC**	**All**	1,717,172	1.9 (-15.3, 19.2)	6.9 (5.9)	79.3	96.9
	**≤12 months**	237,814 (13.8%)	3.9 (-14.3, 22.2)	8.0 (6.0)	72.8	94.8
	**13 to 24 months**	421,465 (24.5%)	4.6 (-13.1, 22.3)	7.9 (6.5)	72.7	95.3
	**25 to 36 months**	416,928 (24.3%)	1.6 (-15.4, 18.6)	6.6 (5.8)	80.7	97.4
	**37 to 48 months**	359,335 (20.9%)	-0.2 (-15.5, 15.2)	6.0 (5.1)	84.8	98.3
	**>48 months**	282,260 (16.4%)	-0.6 (-15.5, 14.3)	5.9 (4.9)	85.3	98.6
**Broselow 2007B**	**All**	1,717,172	5.4 (-15.9, 26.7)	9.4 (7.7)	64.3	91.0
	**≤12 months**	237,814 (13.8%)	4.2 (-19.2, 27.7)	9.6 (7.5)	60.5	88.9
	**13 to 24 months**	421,465 (24.5%)	7.7 (-14.0, 29.3)	10.1 (8.4)	57.7	87.6
	**25 to 36 months**	416,928 (24.3%)	4.9 (-16.2, 25.9)	9.0 (7.6)	66.9	92.3
	**37 to 48 months**	359,335 (20.9%)	4.0 (-15.7, 23.7)	8.4 (6.8)	70.2	94.0
	**>48 months**	282,260 (16.4%)	5.7 (-14.0, 25.5)	9.1 (7.2)	66.0	92.2
**Broselow 2011A**	**All**	1,717,172	7.7 (-13.3, 30.5)	11.2 (8.6)	55.5	85.9
	**≤12 months**	237,814 (13.8%)	6.8 (-26.9, 30.5)	11.0 (8.3)	56.3	85.9
	**13 to 24 months**	421,465 (24.5%)	9.8 (-12.4, 31.9)	12.0 (9.1)	51.6	83.6
	**25 to 36 months**	416,928 (24.3%)	7.6 (-14.0, 29.1)	10.3 (8.4)	59.9	88.6
	**37 to 48 months**	359,335 (20.9%)	8.1 (-12.5, 28.8)	10.5 (8.2)	58.7	87.9
	**>48 months**	282,260 (16.4%)	10.7 (-10.0, 31.4)	12.2 (8.8)	49.9	82.8
**Ralston method**	**No data**					

No subgroup data was available for the Ralston method. The subgroup analyses by age showed the same statistical significance outcomes as for the whole population.

**Table 5:** Weight estimation performance by subgroups of weight.

			MPE (LOA)	RMSPE	p10	p20
**PAWPER XL-MAC**	**All**	1,717,172	1.9 (-15.3, 19.2)	6.9 (5.9)	79.3	96.9
	**≤10kg**	646,539 (37.7%)	6.0 (-11.3, 23.3)	8.3 (5.9)	70.0	94.5
	**10 to 15kg**	906,625 (52.8%)	0.2 (-15.0, 15.3)	6.0 (5.0)	85.2	98.4
	**>15kg**	164,008 (9.6%)	-4.3 (-17.8, 9.2)	6.3 (5.1)	83.2	97.9
**Broselow 2007B**	**All**	1,717,172	5.4 (-15.9, 26.7)	9.4 (7.7)	64.3	91.0
	**≤10kg**	646,539 (37.7%)	9.5 (-11.8, 30.8)	12.0 (7.7)	49.9	83.7
	**10 to 15kg**	906,625 (52.8%)	3.7 (-15.1, 22.4)	7.9 (6.5)	72.7	95.1
	**>15kg**	164,008 (9.6%)	-0.8 (-18.8, 17.2)	7.5 95.6)	74.8	97.3
**Broselow 2011A**	**All**	1,717,172	7.7 (-13.3, 30.5)	11.2 (8.6)	55.5	85.9
	**≤10kg**	646,539 (37.7%)	11.8 (-10.1, 33.7)	13.5 (8.6)	43.3	78.8
	**10 to 15kg**	906,625 (52.8%)	7.2 (-13.3, 27.8)	10.0 (7.9)	61.6	89.2
	**>15kg**	164,008 (9.6%)	3.9 (-14.7, 22.5)	8.3 (6.1)	69.1	95.6
**Ralston method**	**All**	1.800,322	-0.5 (-20.2, 19.3)	Not done	67.4	94.0
	**≤10kg**	677,164 (37.6%)	-1.6 (-23.2, 19.9)	Not done	$\underline{63.2}$	$\underline{92.8}$
	**>10kg**	1,123,158 (62.4%)	0.2 (-18.5, 18.9)	Not done	$\underline{70.5}$	$\underline{96.4}$

Limited subgroup data was available for the Ralston method. The p10 and p20 values were imputed from MPE data (blue underlined type). The subgroup analyses by weight showed the same statistical significance outcomes as for the whole population.

**Table 6:** Weight estimation performance by subgroups of weight status.

			MPE (LOA)	RMSPE	p10	p20
**PAWPER XL-MAC**	**All**	1,717,172	1.9 (-15.3, 19.2)	6.9 (5.9)	79.3	96.9
	**Z≤-2.0**	396,665 (23.1%)	8.9 (-7.4, 25.2)	9.5 (6.8)	63.8	91.5
	**-2.0<Z≤-1.4**	227,073 (13.2%)	4.2 (-9.5, 17.9)	6.3 (5.2)	81.8	98.6
	**-1.4<Z<1.4**	1,025,060 (59.7%)	-0.5 (-16.1, 15.2)	5.7 (5.2)	86.4	99.3
	**1.4≤Z<2.0**	43,122 (2.5%)	-7.3 (-23.0, 8.4)	8.8 (6.3)	64.7	94.5
	**Z≥2.0**	25,252 (1.5%)	-14.4 (-33.9, 5.1)	14.9 (9.3)	36.6	71.8
**Broselow 2007B**	**All**	1,717,172	5.4 (-15.9, 26.7)	9.4 (7.7)	64.3	91.0
	**Z≤-2.0**	396,665 (23.1%)	18.4 (2.3, 34.4)	18.4 (7.8)	13.8	66.1
	**-2.0<Z≤-1.4**	227,073 (13.2%)	10.4 (0.2, 20.6)	10.5 (4.9)	56.0	96.5
	**-1.4<Z<1.4**	1,025,060 (59.7%)	0.7 (-16.3, 17.7)	5.3 (5.8)	88.7	99.8
	**1.4≤Z<2.0**	43,122 (2.5%)	-12.8 (-20.7, -4.8)	12.8 (4.1)	27.9	97.7
	**Z≥2.0**	25,252 (1.5%)	-19.4 (-30.4, -8.3)	19.4 (5.6)	3.5	63.4
**Broselow 2011A**	**All**	1,717,172	7.7 (-13.3, 30.5)	11.2 (8.6)	55.5	85.9
	**Z≤-2.0**	396,665 (23.1%)	22.2 (6.1, 38.4)	22.3 (8.3)	4.6	45.6
	**-2.0<Z≤-1.4**	227,073 (13.2%)	13.9 (4.4, 23.3)	13.9 (4.6)	21.5	92.5
	**-1.4<Z<1.4**	1,025,060 (59.7%)	3.7 (-13.5, 20.8)	6.2 (6.4)	84.1	99.7
	**1.4≤Z<2.0**	43,122 (2.5%)	-10.5 (-17.9, -3.1)	10.5 (3.8)	50.2	99.1
	**Z≥2.0**	25,252 (1.5%)	-17.4 (-28.3, -6.5)	17.4 (5.6)	7.9	75.2
**Ralston method**	**No data**					

No subgroup data was available for the Ralston method for BMI-for-age categories, only for weight-for-height (see below).

**Fig 7:**
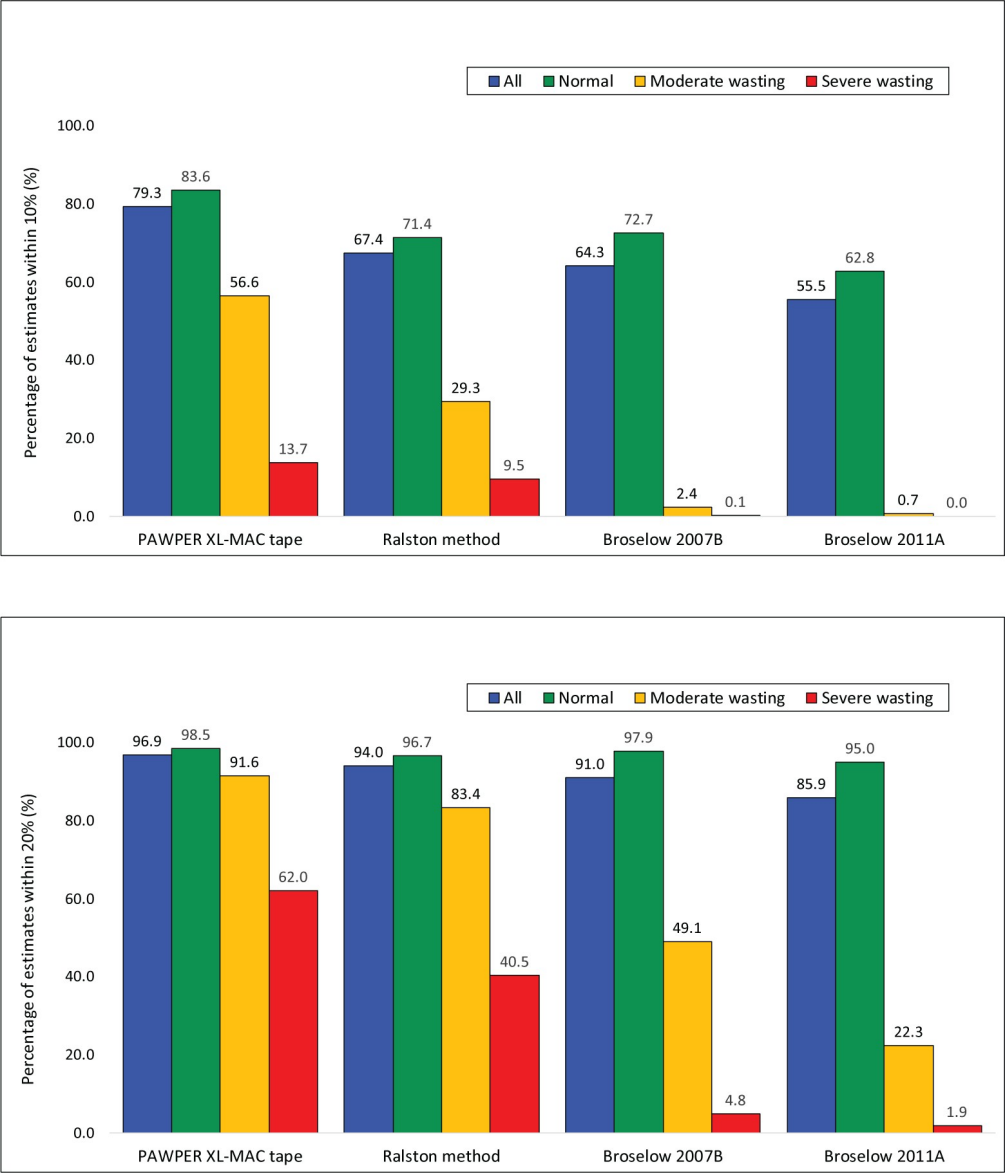
Accuracy outcomes by subgroups of weight-for-height. The p10 data is shown in the upper panel and the p20 data in the lower panel. Children with a weight-for-height Z-score of ≥-2.0 were categorised as “normal”, between -2.0 and -3.0 as “moderately wasted” and below -3.0 as “severely wasted”. The p10 data for the Ralston method was imputed from the MPE data. The McNemar test was significant at the p<0.001 level for every comparison of p10 and p20. We considered the PAWPER XL-MAC method p10 to be clinically superior to all the other methods in all subgroups. The Ralston method p10 was clinically superior to the Broselow tapes in wasted children. The PAWPER XL-MAC method p20 was clinically superior to all other methods in all wasted children. The Ralston method p20 was also clinically superior to the Broselow tapes in all wasted children.

The subgroup analyses of accuracy using weight-for-height cutoffs for the PAWPER XL-MAC and the Ralston method are shown in Fig 7.

When comparing the most and least accurate systems (the PAWPER XL-MAC tape and the Broselow tape 2011 edition A), there was between a 1.4- and 80.4-fold difference in accuracy. This difference was substantially less between the PAWPER XL-MAC tape and the Ralston method ranging between 1.2- and 1.4-fold differences. The full analyses can be found in the Supplementary material (S1 Table).

There were no clinically important differences in the accuracy outcomes between the children from the geographical regions represented in the dataset (Africa, Americas, South-East Asia, Eastern Europe, Middle East and the Western Pacific).

## Discussion

### Performance of the PAWPER XL-MAC system

The PAWPER XL-MAC system performed well in this study and surpassed the predetermined acceptable outcome criteria. It was substantially more accurate than both editions of the Broselow tape as well as the Ralston method (which was originally developed and validated from this dataset). It was also consistently accurate across the geographical regions represented in the dataset, between boys and girls and across the spectrum of age groups and weight categories represented. The PAWPER XL-MAC system was also accurate in all habitus groups except severely underweight and obese children. Despite the lower accuracy at the extremes of habitus, it was substantially and significantly more accurate than the other methods evaluated.

With respect to its accuracy at extremes of habitus, the performance of the PAWPER XL-MAC system was not quite as good in this study as it was in the original validation study [8]. The NHANES dataset used in that study, from a USA population, was markedly different to the one used in this study, however [11]. The children were older (median 120 vs 30 months), heavier (median 40.5 vs 11.1kg) and more overweight (median BMI-for-age Z-score 0.6 vs -0.9). The proportion of “normal” or “average” weight children was also higher (70.6% vs 59.7%). These differences may account for the difference in outcomes. For this system to become truly globally accurate across the age and habitus spectrum, it will need to undergo recalibration or fine-tuning and revalidation to maximise its accuracy (see below). It will also need to be evaluated in prospective studies to determine the impact of human-factor errors and inter-rater reliability.

### Performance of the Broselow tapes

The performance of the Broselow tapes in this dataset was similar to that reported in two recent meta-analyses, which found a p10 range of 50 to 60% to be common [5, 10]. In this study the Broselow tapes were accurate in children with “normal” or “average” weight but were very inaccurate in underweight and overweight/obese children. Since the Broselow tape produces an estimate that more closely approximates ideal body weight (IBW) than total body weight (TBW) this was to be expected [12]. Since IBW may far exceed TBW in underweight children, the use of IBW could result in large drug overdoses in these children [13]. An accurate estimation of TBW is required in all children, irrespective of habitus, to allow accurate drug dosing [13, 14].

The Broselow tape 2007B edition was actually more accurate than the more recent 2011A edition. This was not surprising as the 2011A was the end-result of modifications to the 2007B to reduce its underestimation of weight in children from well-nourished populations with a high prevalence of obesity. This resulted in a worsening of overestimation of weight in children from resource-limited settings. This has also been shown in previous studies from low- and middle-income countries and emphasises the concerns about using the Broselow tape in these settings [15–17].

These findings further highlight the value of the modern two-dimensional (length- and habitus-based) weight estimation systems over one-dimensional (length- or age-based) systems [4]. The Broselow tape should no longer be considered as the “gold standard” in weight estimation. The presence of drug dosing information on the tape is not sufficient to counter its inaccuracy, as this information is incomplete and has not been shown to be beneficial without additional resources [10].

### Performance of the Ralston method

The Ralston method performed reasonably well and was more accurate than the Broselow tapes in the whole sample. It was less accurate than the Broselow tape 2007B in children with “average” weight, but substantially more accurate in underweight children.

The Ralston method was not as accurate as the PAWPER XL-MAC method, including in the subgroups of wasted (underweight) children. Both the PAWPER XL-MAC system and the Ralston method have two categories representing underweight children, although the PAWPER uses the 15^th^ and 5^th^ weight-for-height centiles to define weight categories while the Ralston method uses the 1^st^ and the 5^th^ centiles. More importantly, the PAWPER system uses MAC cut-off values within each length-segment to define habitus while the Ralston method uses the WHO cut-off values for wasting for all children. This enables the PAWPER system to fine tune the weight estimations to a greater degree.

There was no available data on the accuracy of the Ralston method in overweight children. It is likely to be less accurate than both the Broselow 2011A tape and the PAWPER XL-MAC method as it has no mechanism for habitus modification in these children and they are grouped with the “normal” habitus. To be fair, the Ralston method was specifically designed for resource-limited settings, but it must also be remembered that there can still be a significant prevalence of overweight and obese children in low- and middle-income countries [18]. A system that can provide an accurate weight estimation for a wide range of children is therefore required. Further work on the Ralston method may produce useful information to advance length- and habitus-based weight estimation.

### Other methods

The Mercy method makes use of humeral length (as a surrogate for body length) and MAC to generate a weight estimate [19]. Some previous studies have shown the Mercy method to be very accurate in populations with a high prevalence of young and underweight children [20, 21], while others have found it to be less accurate in these children [17]. Given its high level of accuracy, and the fact that it has been promoted for use in resource-limited areas where scales are not available or might be poorly calibrated, it would have been useful to compare it with the PAWPER XL-MAC and Ralston methods in this study [1]. Unfortunately, it could not be evaluated in this dataset as humeral length was not available.

### The “sophistication of simplicity”

In order for a weight estimation system to be successful, it must be accurate and easy to use: the “sophistication of simplicity” [12]. The PAWPER XL-MAC system fulfils these requirements as it makes use of straightforward, reliable anthropometric parameters from which a weight estimation can be read directly off the tape, without requiring any calculations. Both the PAWPER XL tape and Mercy methods have been tested in simulated paediatric resuscitations under adverse, clinically realistic circumstances and have been shown to be resilient (i.e. maintain their accuracy) [22]. The use of MAC measurements is therefore reasonable and appropriate as part of the weight estimation process, whether it is during an emergency when a child cannot be weighed or in an environment where a scale is not available.

### The need for recalibration

Although the PAWPER XL-MAC system achieved the predetermined acceptable outcome criteria, its performance in severely underweight and obese children was not as good as in other habitus types. A previous study with the original PAWPER tape in an obese USA population had similar findings, even though the tape was still the most accurate of the methods evaluated. It is not yet clear whether the inherent biological variability in the relationship between MAC and body habitus will allow an increase in weight estimation accuracy using this methodology. Nonetheless, the possibility of recalibrating the corrected weights or the MAC cut-off values in the upper habitus categories needs to be explored in future versions of the tape.

### Limitations

One of the major limitations of every weight estimation study is that it is not known what degree of accuracy is required by a weight estimation system to prevent patient harm resulting from drug dosing errors. Although we regard a p10 of 70% and a p20 of 95% as an indicator of acceptable outcome, this is based on expert speculation only.

The second limitation of this study is that this was a “virtual” study with anthropometric data obtained from a database rather than from the tape actually being used in clinical practice. This provides very useful information on the potential accuracy of the weight estimation systems but does not provide evidence on human- and patient-factor errors and inter-user reliability. However, the difference between “virtual” and “in real life” testing has never been evaluated and it is not clear whether there would be a substantial difference in outcomes.

Finally, the need to impute some of the subgroup accuracy data for the Ralston method may have influenced the statistical analysis. This was not likely to have altered any of the findings to any significant degree, however.

## Conclusions

The PAWPER XL-MAC tape was the most accurate of the weight estimation systems evaluated. It achieved the acceptable outcome criteria in the dataset of children from low- and middle-income countries as a whole and in all subgroups except children at the extremes of habitus. Even in these subsets it outperformed the other systems. The tape needs to be validated in prospective studies to establish whether this accuracy can be maintained in clinical practice.

The Ralston method showed promise, especially in profoundly wasted children, but needs to be evaluated further, especially to see whether its methodology can be sustained in real-world situations.

Both editions of the Broselow tape performed worse than the two-dimensional methods. The Broselow tape should no longer be regarded as a “gold standard” method.

## Supporting information

S1 File The PAWPER XL-MAC formula. This Microsoft Excel file contains the formula that can generate a weight estimate using recumbent length and mid-arm circumference measurements based on the PAWPER XL-MAC method.
(XLSX)

S1 Table Outcomes of comparisons between the PAWPER XL-MAC method p10 and the other weight estimation systems. This tables shows the details of the comparisons in accuracy (p10) between the PAWPER XL-MAC tape and the other methods evaluated.
(DOCX)
