# Supplementary material for: A validation of the PAWPER XL-MAC tape for total body weight estimation in preschool children from low- and middle-income countries
Source: PLoS One. 2019 Jan 7;14(1):e0210332. doi: 10.1371/journal.pone.0210332 (PMC6322773; doi:10.1371/journal.pone.0210332)
Supplement: S1 Table — This tables shows the details of the comparisons in accuracy (p10) between the PAWPER XL-MAC tape and the other methods evaluated. (DOCX) [file pone.0210332.s002.docx]

|  |  | 2011A | 2007B | Ralston |
| --- | --- | --- | --- | --- |
| All | Absolute difference (%) | 24 (24, 25) | 15 (14, 16) | 12 (12, 13) |
|  | Relative difference (%) | 43 (42, 44) | 23 (22, 24) | 18 (18, 19) |
|  | Odds ratio (95%CI) | 4.4 (4.4, 4.5) | 2.9 (2.8, 2.9) | 1.8 (1.8, 1.8) |
|  | P value | <0.001 | <0.001 | <0.001 |
| Z<=-2 | Absolute difference (%) | 59 (57, 61) | 50 (48, 52) | This subgroup data was not available for the Ralston method. |
|  | Relative difference (%) | 1280 (1265, 1640) | 362 (340, 390) |  |
|  | Odds ratio (95%CI) | 51 (50, 52) | 15 (14, 15) |  |
|  | P value | <0.001 | <0.001 |  |
| -1.4<=Z<-2 | Absolute difference (%) | 60 (56, 68) | 26 (24, 30) |  |
|  | Relative difference (%) | 281 (276, 302) | 46 (42, 54) |  |
|  | Odds ratio (95%CI) | 22 (21, 23) | 5.0 (4.9, 5.1) |  |
|  | P value | <0.001 | <0.001 |  |
| -1.4<Z<1.4 | Absolute difference (%) | 2 (2, 2) | -2 (-2, -2) |  |
|  | Relative difference (%) | 3 (3, 3) | -3 (-3, -3) |  |
|  | Odds ratio (95%CI) | 1.7 (1.6, 1.8) | 0.8 (0.8, 0.8) |  |
|  | P value | <0.001 | <0.001 |  |
| 1.4<=Z<2.0 | Absolute difference (%) | 14 (13, 15) | 28 (27, 29) |  |
|  | Relative difference (%) | 29 (25, 35) | 78 (74, 88) |  |
|  | Odds ratio (95%CI) | 2.5 (2.5, 2.5) | 4.5 (4.4, 4.5) |  |
|  | P value | <0.001 | <0.001 |  |
| Z>=2 | Absolute difference (%) | 29 (28, 30) | 33 (31, 37) |  |
|  | Relative difference (%) | 360 (290, 454) | 940 (860, 1560) |  |
|  | Odds ratio (95%CI) | 9.4 (9.3, 9.5) | 22 (22, 22) |  |
|  | P value | <0.001 | <0.001 |  |
|  |  |  |  |  |
| WHZ <-3 | Absolute difference (%) | 14 (12, 15) | 14 (13, 14) | 4 (4, 4) |
|  | Relative difference (%) | 618414 | 9415 | 45 (42, 52) |
|  | Odds ratio (95%CI) | 7170 (1010, 50909) | 280 (183, 424) | 1.7 (1.6, 1.9) |
|  | P value | <0.001 | <0.001 | <0.001 |
| WHZ -2 to -3 | Absolute difference (%) | 56 (55, 58) | 54 (52, 60) | 27 (35, 30) |
|  | Relative difference (%) | 8180 (7468, 12604) | 2240 (2068, 2846) | 93 (85, 128) |
|  | Odds ratio (95%CI) | 281 (251, 314) | 57 (54, 60) | 2.1 (2.0, 2.1) |
|  | P value | <0.001 | <0.001 | <0.001 |
| WHZ>=-2 | Absolute difference (%) | 21 (20, 23) | 11 (10, 13) | 8 (8, 8) |
|  | Relative difference (%) | 33 (31, 35) | 15 (14, 17) | 17 (16, 18) |
|  | Odds ratio (95%CI) | 3.6 (3.6, 3.6) | 2.2 (2.2, 2.3) | 1.9 (1.9, 1.9) |
|  | P value | <0.001 | <0.001 | <0.001 |

**S1 Table. Outcomes of comparisons between the PAWPER XL-MAC method p10 and the other weight estimation systems.**

Odds ratio interpretation

≈6.0 large effect size

≈3.5 moderate effect size

≈1.5 small effect size
